# Positron Range Correction Helps Enhance the Image Quality of Cardiac ^82^Rb PET/CT

**DOI:** 10.2967/jnumed.124.267855

**Published:** 2025-03

**Authors:** Martin Lyngby Lassen, Hunor Kertész, Ivo Rausch, Vladimir Panin, Maurizio Conti, Sven Zuehlsdorff, Jorge Cabello, Deepak Bharkhada, Robert DeKemp, Andreas Kjaer, Thomas Beyer, Philip Hasbak

**Affiliations:** 1 Department of Clinical Physiology and Nuclear Medicine, University Hospital Copenhagen–Rigshospitalet, Copenhagen, Denmark;; 2 Cluster for Molecular Imaging, Department of Biomedical Sciences, Faculty of Health and Medical Sciences, University of Copenhagen, Copenhagen, Denmark;; 3 Image X Institute, Faculty of Medicine and Health, University of Sydney, Sydney, New South Wales, Australia;; 4 QIMP Team, Medical University of Vienna, Vienna, Austria;; 5 Siemens Medical Solutions USA, Inc., Knoxville, Tennessee; and; 6 National Cardiac PET Centre, University of Ottawa Heart Institute, Ottawa, Ontario, Canada

**Keywords:** cardiac PET, myocardial perfusion imaging, ^82^Rb, positron range correction

## Abstract

The image quality and quantitative accuracy of ^82^Rb myocardial perfusion imaging (MPI) using PET is challenged by the extensive positron range (PR) effects, with the PR of ^82^Rb being about 7 mm in soft tissues. This study explored the feasibility of applying postacquisition PR correction (PRC) to routine ^82^Rb PET/CT MPI acquisitions and assessed its impact on diagnostic accuracy and image quality. **Methods:** We implemented a PRC method adjusted to ^82^Rb into a vendor-provided reconstruction toolbox, using tissue-specific corrections for soft tissue, bone, and air/lungs. The PRC was evaluated in 2 cohorts: the first comprised 25 healthy volunteers who underwent repeated ^82^Rb MPI within 2 wk, and the second included 66 patients with known or suspected coronary artery disease. We measured the signal-to-noise ratio (SNR) and contrast-to-noise ratio (CNR) for the volunteer cohort. In the patient cohort, the impact of PRC was evaluated as changes in the area under the receiver operating characteristic curve (AUC), using fractional flow reserve as the gold standard (values < 80% were considered significantly reduced). We calculated AUCs for stress and ischemic total perfusion deficits. **Results:** In the volunteer cohort, PRC-based reconstructions (standard reconstruction [STD] + PRC) demonstrated significantly improved SNR and CNR compared with STD, with median increases of 22% and 47% for SNR and CNR, respectively (*P* < 0.05). For the patient cohort, comparable AUCs were reported for STD- versus PRC-based reconstructions (stress total perfusion deficits, 0.84 vs. 0.83 [*P* = 0.49]; ischemic total perfusion deficits, 0.87 vs. 0.87 [*P* = 0.80]). **Conclusion:** PRC significantly enhances SNR and CNR compared with STD without affecting the diagnostic accuracy of the scans. Given the significantly improved image quality, PRC may be recommended for MPI using ^82^Rb PET/CT clinical-routine-assessment interpretation of TPD.

## Visual Abstract

**Figure:**
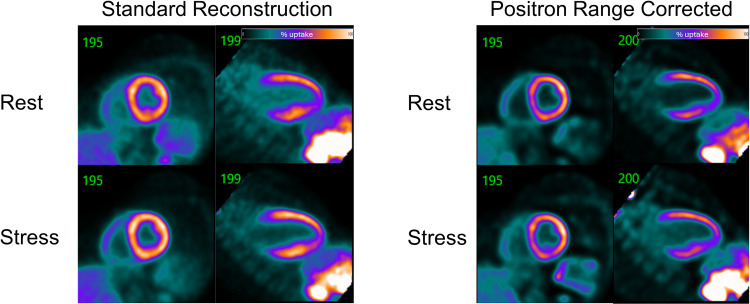

Myocardial perfusion imaging (MPI) with ^82^Rb is the predominant myocardial PET examination, offering the advantage of assessing relative perfusion, absolute blood flow, and function without the need for an on-site cyclotron (1,2). Despite its well-established diagnostic and prognostic capabilities (1,3–6), ^82^Rb comes with challenges for PET imaging, namely its long positron range (PR) (in soft tissue, mean PR of ∼7 mm and maximum PR of ∼17 mm) (3–5). The mean PR in water for other frequently used PET tracers is considerably shorter (^18^F, ∼0.6 mm; ^64^Cu, ∼0.7 mm; and ^68^Ga, ∼2.3 mm) (5,6). The long PR of ^82^Rb degrades PET image quality through reduced contrast between the blood pool and left ventricular wall and, potentially, between myocardial tissues with and without perfusion defects.

Recent advancements have introduced a clinical retrospective PR correction (PRC) method, integrated into vendor-provided reconstruction software. The PRC method has been tested in phantom and oncologic studies involving high-energy positron-emitting radionuclides such as ^68^Ga and ^124^I (7). Given the significance of ^82^Rb in cardiac PET imaging, the implementation of PRC in routinely acquired MPI is worth investigating both quantitatively and qualitatively. In this study, PRC was tested and validated in a volunteer cohort and a cohort of patients with known or suspected perfusion defects. The evaluation of the PRC included assessments of signal-to-noise ratio (SNR) and contrast-to-noise ratio (CNR) and changes in diagnostic performance using the fractional flow reserve (FFR) as the gold standard.

## MATERIALS AND METHODS

### Study Population

Two study populations were considered: healthy volunteers and patients with an intermediate to high risk of cardiovascular disease referred for coronary angiography with or without known coronary artery disease.

The volunteer cohort comprised 25 young individuals who underwent 2 ^82^Rb rest/adenosine stress MPI sessions within 2 wk, adhering to specific inclusion and exclusion criteria as described in a previous study (8). The Scientific Ethics Committee (institutional review board) (protocol H-15009293) and the Danish Data Protection Agency approved this study, and all subjects gave written informed consent.

The other cohort included 66 patients referred for coronary angiography and ^82^Rb rest/adenosine stress MPI because of chronic coronary syndromes (Table 1). This study was approved by the Capital Region of Denmark (institutional review board) (protocol H-42014046), as well as the Danish Data Protection Agency, and all subjects gave written informed consent. Of the 66 patients, 27 had known coronary artery disease and 39 had no known coronary artery disease. For this cohort, the sole inclusion criterion was age greater than 50 y, whereas the exclusion criteria were claustrophobia, severe asthma, or renal failure (plasma creatinine > 140 μM). Coronary stenoses greater than 50% identified by invasive angiography were, in all cases, assessed for hemodynamic significance by measuring FFR during a continuous adenosine infusion (140 µg/kg/min) for 2 min. An FFR of less than 0.8 was considered significant and led to subsequent percutaneous coronary intervention when technically possible. In 34 of the 64 patients, percutaneous coronary intervention was deemed necessary after ^82^Rb and FFR assessments.

**TABLE 1.** Patient Demographics

Demographic	No known coronary artery disease (*n* = 39)	Known coronary artery disease (*n* = 27)
Age (y)	64 (58–70)	66 (58–71)
Female	10 (26%)	8 (30%)
Body mass index (kg/m^2^)	28 (24–33)	29 (26–30)
1-, 2-, and 3-vessel disease	7, 3, and 5	0, 14, and 13
Left ventricular ejection fraction		
Rest	67 (58–71)	55 (41–62)
Stress	69 (62–75)	58 (41–63)
Current smoking	8 (21%)	4 (15%)
COPD	6 (15%)	6 (22%)
Diabetes mellitus	7 (18%)	6 (22%)
Hypertension	22 (56%)	22 (7%)
Hypercholesterolemia	26 (67%)	22 (81%)
Family history of IHD	14 (36%)	13 (48%)
History of myocardial infarction	0 (0%)	10 (37%)
Previous PCI	0 (0%)	9 (33%)
Previous CABG	0 (0%)	4 (15%)

COPD = chronic obstructive pulmonary disease; IHD = ischemic heart disease; PCI = percutaneous coronary intervention, CABG = coronary artery bypass grafting.

Qualitative data are number and percentage; continuous data are median and interquartile range.

### Imaging Protocol

All study participants underwent rest/adenosine stress MPI using a 128-slice Biograph mCT PET/CT system (Siemens Healthineers) using targeted injection doses of 1,100 MBq of ^82^Rb. Stress MPI was achieved using adenosine infused intravenously at a rate of 140 µg/kg/min for 6 min, with a PET emission acquisition starting 2.5 min into the adenosine infusion. Low-dose CT, for attenuation correction of the PET emission data, was performed in an end-expiratory breath-hold before the PET acquisition (120 kVp; effective tube current, 26 mA [11-mAs quality reference]; pitch, 1.5, with an effective dose of 0.4 mSv) (9). The low-dose CT and PET emission data were coregistered before PET image reconstructions to minimize the risks of misalignment-induced artifacts in the images (10). Caffeine abstinence was required for at least 16 h before each imaging session.

### PRC

PRC was implemented into a vendor-based image reconstruction framework (e7-tools; Siemens Healthineers) as an additional PR-dependent point-spread function correction, as previously described by Kertész et al. (6,7). In brief, the PR distributions of ^82^Rb in 3 different tissues (density in lung, 0.26 g/cm^3^; density in water, 1.00 g/cm^3^; and density in bone, 1.92 g/cm^3^) were obtained using GATE 9.0 (GEANT4 10.06.p02) Monte Carlo simulations. Uniform PR kernels were calculated by mapping the PR distribution to a voxel grid given by the maximum PR in water and the voxel size of the PET system. From these PR distributions, a spatially variant and tissue-dependent PR distribution (PR kernel) was generated for every voxel by combining the uniform PR kernels after an analysis of the underlying tissue composition obtained from the attenuation correction map. The kernels are applied to the current image estimate as a convolution in image space before the forward-projection step (6,7).

### Image Reconstruction

Two image series were considered: one using the standard vendor-provided protocol (STD) and the other the proposed PRC-based protocol (STD + PRC) (7). Both protocols included emission data obtained between 150 and 360 s after injection and used corrections for time of flight and the system-specific point-spread function (400 × 400 × 109 matrix; 2.04 × 2.04 × 2.07 mm voxel size). For STD, we used 2 iterations and 21 subsets. To achieve comparable convergence with STD + PRC, we used 3 iterations and 21 subsets. Determination of these parameters was based on visual assessments by a physician with over 15 y of experience analyzing ^82^Rb PET images and aligned with previous reports suggesting that STD + PRC needs more iterations to achieve similar convergence to STD (7). Importantly, the choice of iterations was based on reconstructions using 1–7 iterations, as shown in Supplemental Figure 1 (supplemental materials are available at http://jnm.snmjournals.org). Both image series were filtered using a 5-mm gaussian filter. No actions were taken toward introducing motion correction into the image reconstruction of either reconstruction protocol.

### Image Analysis

**FIGURE 1.**
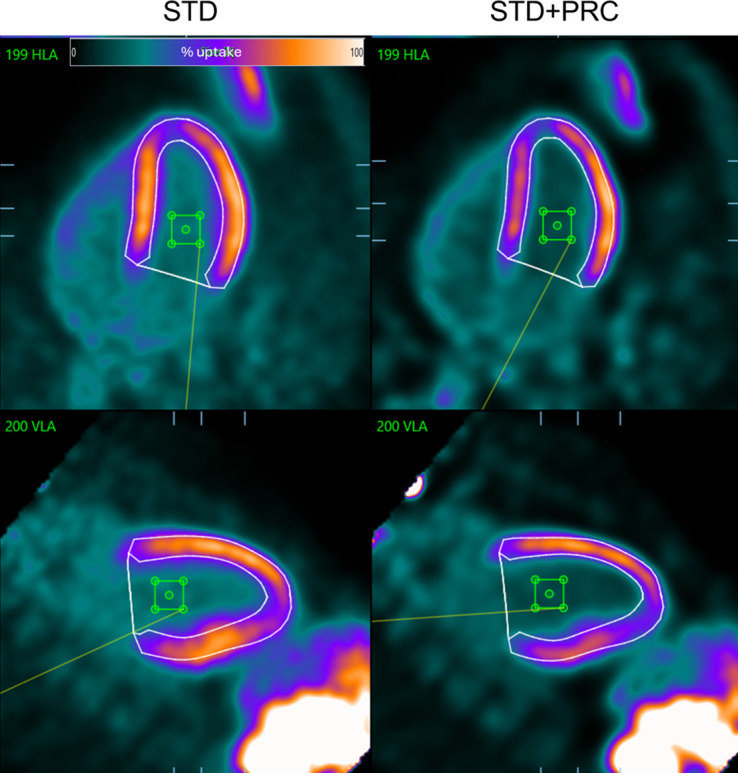
Visualization of myocardial segmentation and 15 × 15 × 15 mm volume of interest (green box) inserted in left ventricular lumen. Percentage uptake is relative to uptake within segmented left ventricle. HLA = horizontal long axis; VLA = vertical long axis.

All images were assessed using QPET software (Cedars-Sinai) in a masked fashion by an experienced reader (with >15 y of experience). For the volunteer cohort, we report the SNR, CNR, and noise in the blood pool measured as the coefficient of variation. In brief, the SNR was calculated using the SUV_mean_ derived from the LV polar map, divided by the SD of the SUV_mean_ of the blood pool (using a 15 × 15 × 15 mm volume of interest inserted in the left ventricular lumen using MATLAB [MathWorks]) (Fig. 1). QPET offers SUV_mean_ measurements for each segment of the polar plots. Likewise, the CNR was calculated as the [SUV_mean_ (polar map) – SUV_mean_ (blood pool)]/SD of the SUV (blood pool). In contrast to the more frequently used spheric volumes of interest in oncologic settings, we used cylindric volumes of interest in this nuclear cardiology study. By doing so, we adhered to the elongated shape of the heart and reduced the risk of spill-in effects from the myocardial wall and motion-induced partial-volume effects. The apparent changes in myocardial uptake in all figures visualizing STD + PRC may indicate a falsely reduced uptake in the myocardium, likely caused by increased extracardiac focal uptake elsewhere in the image.

For the patient cohort, we report the total perfusion deficits for all reconstructed datasets (rest total perfusion deficit and stress total perfusion deficit [sTPD]) and the ischemic total perfusion deficit (iTPD), calculated as sTPD minus rest total perfusion deficit, for the individual MPI sessions (10,11).

### Custom Normal Databases

PRC significantly impacted data distribution, introducing redistribution of the respective uptake in both the myocardial wall and the surrounding tissues, thus inducing an imperative need for new normal databases to assess the potential perfusion defect size and severities. To this end, we generated 2 new normal databases, one for STD and the other for STD + PRC, both based on the test studies in the healthy volunteer cohort. This cohort comprised young, healthy individuals without any preexisting cardiovascular disease or lifestyle-induced adverse effects on the cardiovascular system, thus providing a unique normal database. The STD normal database was expected to mirror the characteristics outlined by Nakazato et al. (12). Conversely, STD + PRC was anticipated to maintain the same underlying quality and functionality, accounting for the PRC corrections in STD + PRC images. The custom normal databases were tested on the retest scans (volunteer cohort) to validate their stability and accuracy. The introduction of the 2 custom normal databases had a second benefit, as it permitted head-to-head comparison of the estimated TPDs.

### Statistical Analysis

The data were tested for normality using the Shapiro–Wilk test. Continuous data are presented as mean ± SD or as median and interquartile ranges, and categoric data are presented as percentages. Using the FFR findings for the patient population, we evaluated the impact of PRC through receiver operating characteristic curves in per-patient and per-vessel assessments. All receiver operating characteristic curves were compared using the DeLong method (13), using the area under the receiver operating characteristic curve (AUC) as the primary endpoint for this study. The sensitivity and specificity of the 2 reconstruction methods were calculated using a 2 × 2 confusion matrix, using the optimal threshold of the receiver operating curve for both STD and STD + PRC. McNemar tests were performed to test the sensitivity and specificity of the 2 reconstruction protocols (14). The normalcy of the study was defined as the number of healthy volunteers who had TPDs below the recommended thresholds for subsequent FFR assessments. Finally, SNR and CNR test–retest repeatability was calculated for the healthy volunteers using the intraclass correlation coefficient, with the Fisher Z-transform being applied to test for statistical differences between the 2 reconstruction protocols. The Fisher Z-transform changes the sampling distribution to become normally distributed, which is used to create 95% CIs (15).

## RESULTS

### Image Quality (Volunteer Cohort)

**FIGURE 2.**
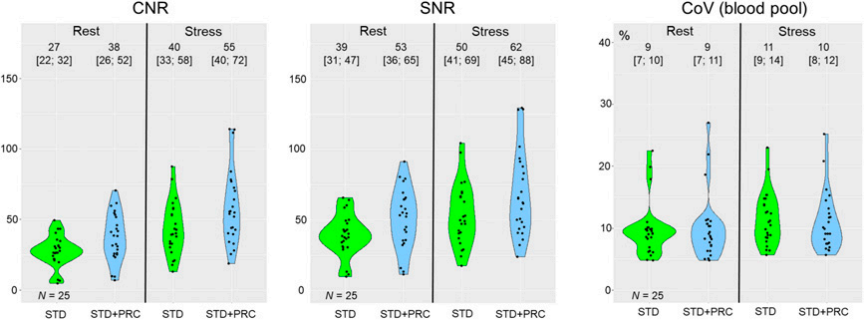
Comparison of SNR and CNR in left ventricle and noise in blood pool (coefficient of variation) obtained for STD and STD + PRC images in 25 healthy volunteers. PRC improves SNR and CNR significantly in both rest and stress conditions. In contrast, noise in blood pool is preserved, suggesting that increased activity recovery in myocardial wall drives improvements in SNR and CNR. Numbers above each violin plot indicate median and interquartile range of measurements. CoV = coefficient of variation.

**FIGURE 3.**
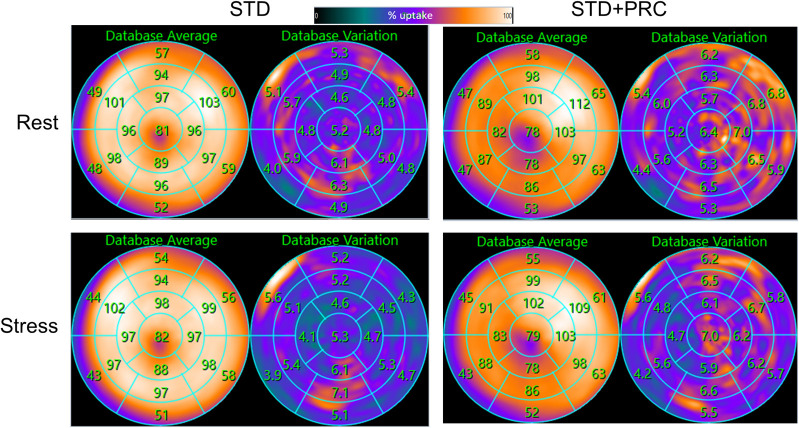
Mean and SD of normal databases for STD and STD + PRC. Anterior/inferior gradient is observed in normal database of 25 healthy volunteers. Reduced perfusion in apical region for STD + PRC may reflect generally reduced perfusion in this area because of thin wall.

STD + PRC significantly improved the SNR and CNR when the global left ventricular wall was evaluated (improvements ≥ 25% of values obtained using STD, all *P* < 0.05), whereas the background noise obtained in the blood pool (measured as the coefficient of variation) was preserved (*P* = 0.36 for rest and 0.29 for stress) (Fig. 2). Test–retest repeatability for the SNR and CNR was comparable for both STD and STD + PRC, with global intraclass correlation coefficients ranging between 0.27 and 0.41 (STD) and 0.35 and 0.36 (STD + PRC) for the global assessments (all *P* ≥ 0.70) and confirmed by the Fisher Z-test with *P* values in the range 0.17–1.00 (Table 2). Comparisons of the mean and SD of the polar maps for the normal databases are shown in Figure 3, demonstrating a gradient bias for STD + PRC, with the highest uptake in the anterior wall. Despite differences in the uptake patterns across the 2 image reconstruction protocols, the custom normal databases provided comparable sTPD and iTPD assessments of retest scans for the volunteer cohort (**Table 3**).

**TABLE 2.** Test–Retest Repeatability for SNR and CNR Obtained for STD- and PRC-Based Reconstructions

Parameter	LAD	LCx	RCA	Global
SNR				
STD, rest	0.33 (−0.08–0.66)	0.31 (−0.10–0.65)	0.29 (−0.14–0.63)	0.39 (−0.03–0.70)
STD + PRC, rest	0.41 (−0.47–0.72) (*P* = 0.58)	0.54 (0.11–0.79) (*P* = 0.17)	0.36 (−0.08–0.69) (*P* = 0.70)	0.36 (−0.12–0.70) (*P* = 0.70)
STD, stress	0.41 (0.01–0.71)	0.35 (−0.09–0.68)	0.45 (0.04–0.73)	0.35 (−0.03–0.70)
STD + PRC, stress	0.34 (−0.04–0.66) (*P* = 0.69)	0.33 (−0.05–0.65) (*P* = 0.65)	0.32 (−0.06–0.64) (*P* = 0.45)	0.35 (−0.10–0.68) (*P* = 1.00)
CNR				
STD, rest	0.35 (−0.06–0.66)	0.32 (−0.10–0.65)	0.29 (−0.14–0.63)	0.27 (−0.17–0.62)
STD + PRC, rest	0.43 (−0.02–0.73) (*P* = 0.65)	0.55 (0.14–0.80) (*P* = 0.22)	0.35 (−0.10–0.68) (*P* = 0.75)	0.35 (−0.01–0.69) (*P* = 0.75)
STD, stress	0.43 (0.03–0.72)	0.36 (−0.08–0.68)	0.48 (0.07–0.75)	0.41 (−0.01–0.71)
STD + PRC, stress	0.29 (−0.08–0.62) (*P* = 0.43)	0.29 (−0.08–0.62) (*P* = 0.70)	0.27 (−0.09–0.59) (*P* = 0.23)	0.35 (−0.11–0.67) (*P* = 0.73)

LAD = left anterior descending coronary artery; LCx = left circumflex coronary artery; RCA = right coronary artery.

Data are intraclass correlation coefficients followed by 95% CI. *P* values were obtained from Fisher Z-test (test for differences across 2 reconstruction methods).

**TABLE 3.** sTPD and iTPD for Retest Scans Using 2 Reconstruction Protocols in Volunteers

Parameter	STD	STD + PRC
sTPD	2 ± 2	2 ± 1
iTPD	2 ± 2	2 ± 1

Normal databases were generated from test scans, and their efficacy was tested on retest scans.

### Receiver Operating Curve Analysis (Patients)

**FIGURE 4.**
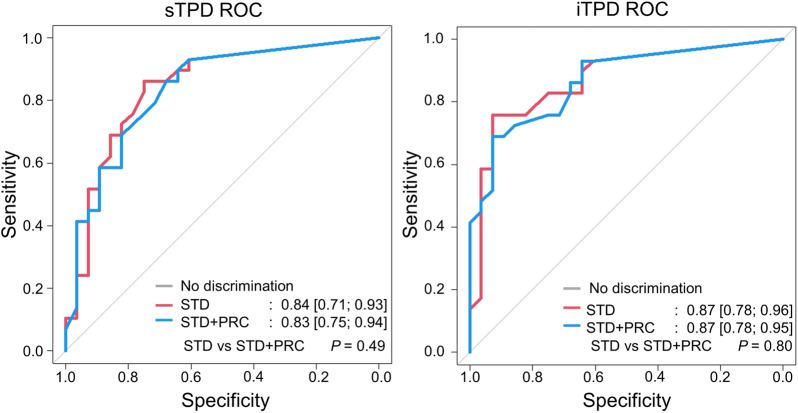
sTPD and iTPD analyses of 66 patients obtained using STD and STD + PRC. There was no difference in AUC between protocols. 95% CIs are provided in brackets. ROC = receiver operating characteristic curve.

**FIGURE 5.**
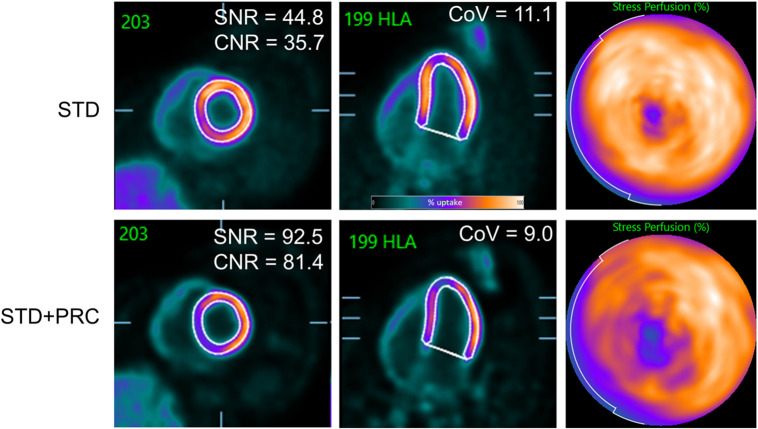
Example of stress MPI of volunteer. STD + PRC images had significantly increased SNR and CNR compared with STD. Noise levels in blood pool remained, indicating that improved SNR and CNR metrics were obtained solely by improved activity recovery in myocardial wall. This contrast improvement was evident as relative reduction in blood-pool activity within left and right ventricles, whereas activity in left ventricular wall was less dispersed. CoV = coefficient of variation (measured in blood pool). HLA = horizontal long axis.

**FIGURE 6.**
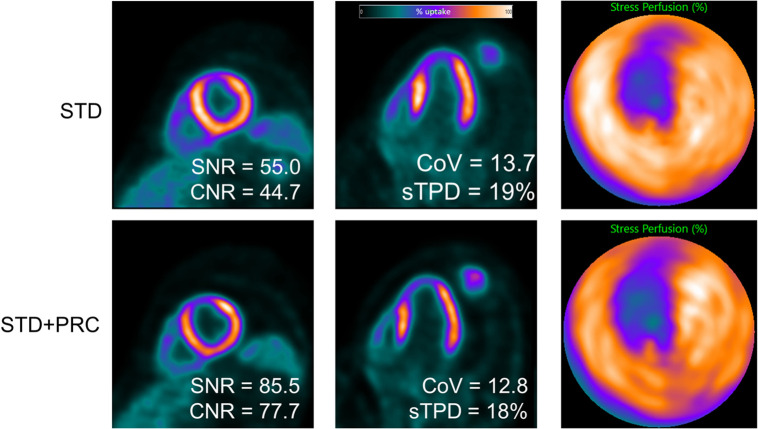
Example of stress MPI of patient with FFR-positive stenosis in LAD. After application of PRC, perfusion defect in anterior wall of left ventricle became more pronounced than what was observed for STD, even though sTPD values remained equivocal. Similarly to Figure 4, PRC notably enhanced SNR and CNR while maintaining consistent noise properties within left ventricular lumen. CoV = coefficient of variation (measured in blood pool).

Per-patient assessments of AUC obtained for STD and STD + PRC appeared to have similar distributions and, thus, suggest equivocal performance of the 2 reconstruction methods (iTPD: STD = 0.87, STD + PRC = 0.87, *P* = 0.80) (Fig. 4). Per-vessel AUC for the reconstruction protocols are shown in Supplemental Figure 2. Although no statistical difference in the AUC was observed, the STD + PRC images showed a significantly improved visual quality (Figs. 5 and 6). Recommended thresholds for STD and STD + PRC were extracted from the receiver operating curves: 9% for sTPD and 8% for iTPD, and 8% for sTPD and 10% for iTPD, respectively. Using these thresholds, the normalcy rate was 100% for both sTPD and iTPD for both reconstruction protocols; that is, all healthy subjects were correctly identified. Further, the sensitivities, specificities, and positive and negative predictive values were comparable in performance for the 2 reconstruction protocols along McNemar tests for sensitivity and specificity (Table 4). Thirty-four of the 64 patients proceeded with percutaneous coronary intervention after the ^82^Rb imaging and subsequent coronary angiography with FFR assessments.

**TABLE 4.** Sensitivity and Specificity for sTPD and iTPD for 2 Reconstruction Protocols

	sTPD		iTPD	
Parameter	STD	STD + PRC	STD	STD + PRC
Sensitivity	83.9 (59.0–87.2)	80.6 (56.8–86.3)	74.2 (75.0–97.8)	71.0 (72.2–97.5)
Specificity	77.1 (65.6–91.4)	74.3 (60.9–87.9)	94.3 (65.9–89.8)	80.0 (60.6–85.4)
Positive predictive value	75.8	74.2	92.0	90.9
Negative predictive value	81.8	77.1	80.5	75.0
McNemar test		*P* = 0.617		*P* = 0.248

Data in parentheses are 95% CI.

## DISCUSSION

This study investigated the effects of applying PRC to routinely acquired ^82^Rb PET MPI. Cardiac ^82^Rb PET images display lower contrast than ^13^N-NH_3_ and ^18^F-flurpiridaz images (16), as is attributed in part to the lower energy of positrons emitted by ^13^N and ^18^F. The main finding of our study was that PRC can be implemented for routine ^82^Rb PET-based MPI as a retrospective correction, thereby requiring only the routinely acquired low-dose CT attenuation correction maps and the PET raw data (list-mode file). The proposed PRC method improved the SNR and CNR between the left ventricular wall and lumen without altering the underlying noise in the blood pool (background). Finally, whereas PRC did not appear to change the AUC, it significantly improved visual assessments of ^82^Rb MPI data.

This study successfully tested a PRC method incorporating corrections during the forward projection for each subset and each iteration during the iterative PET image reconstruction algorithm. Here, the PRC method considered 3 tissue types (soft tissue [water], bone, and air [lungs]), which were segmented from the low-dose CT attenuation correction maps. Because of differences in ^82^Rb PRs among these tissue types, a spatially dependent bias was observed when comparing the anterior walls (neighboring lung tissue) and inferior walls (neighboring soft tissues) of the left ventricle of the heart (Figs. 2 and 4). To alleviate the spatially dependent variance for STD + PRC, 2 custom normal databases were created from the test scans of the volunteer cohort, one seeking to provide normal values for the STD and the other for the STD + PRC image series (Fig. 2).

In brief, the custom normal databases created for this study provided a unique assessment of normality given the young, healthy subjects. Further, the custom normal databases permitted a head-to-head comparison of the mean activity distribution for the 2 normal databases, which revealed several striking differences. First, a gradient (anterior/posterior direction) for STD + PRC was observed in the normal database, with the highest activity distributions observed in the anterior segment; in contrast, STD provided a homogeneous activity distribution throughout the entire left ventricle (Fig. 2). Second, the apical region appeared less perfused in the PRC images, as may be attributed to a better activity recovery (and thereby delineation) of this segment, which generally is difficult to encompass because of the displacement of the apex during the cardiac cycle.

Comparisons of the performance of normal databases for STD and STD + PRC revealed no differences for either image series despite the significant changes in underlying image quality. Finally, the SD for STD + PRC was compared with that for STD (Fig. 3). The observed differences may be explained by an increased sensitivity to motion during STD + PRC and heterogeneous injection profiles throughout the generator lifespan (17,18). Unfortunately, these hypotheses cannot be tested further, given the relatively small cohorts evaluated in this study. In addition, more extensive comparisons of normal databases were beyond the scope of this study, which focused primarily on assessing the feasibility of using PRC in routine ^82^Rb MPI.

Despite the relatively small sizes of our cohorts, we observed significantly elevated SNR and CNR in both the volunteer and the patient cohorts with STD + PRC. In addition, no differences in AUC were observable for STD + PRC, thus suggesting the 2 reconstructions to be equivalent (Figs. 1 and 3; Tables 2 and 4). The per-vessel receiver operating curves, and accompanying AUCs, were significantly improved compared with the per-patient analyses. This was due to a 3-fold increase in number of measurements (3 vessels) whereas the number of coronary artery stenosis measurements remained constant (Supplemental Fig. 2). These results were due to the FFR measurements that were restricted to vessels with visible stenosis according to institutional guidelines and omitted coronary arteries assumed to have less than a 50% stenosis diameter (which were treated as observations with a value of 0 in the subsequent AUC analyses).

Naturally, several factors, such as shifts in the heart during the PET emission acquisition (myocardial motion, breathing, and myocardial creep), may further affect PRC performance. Recent research from our center has identified that myocardial creep and cardiorespiratory motion affect AUC assessments, particularly in patient cohorts similar to those in this study (19). The motion-induced shift in the left ventricular wall (and concomitant nonlinear changes in tissue distributions) will reduce the activity recovery of PRC after processing. Unfortunately, combining motion correction with PRC in the current implementation would pose significant challenges because of the requirement for a minimum of 4 gates for single-gate motion correction (either cardiac or respiratory), 16 gates for dual-gating approaches (4 cardiac and 4 respiratory phases), and at least 32 gates for a triple-corrected dataset (cardiorespiratory motion correction with 1 creep or reposition event during the acquisition) (19). Therefore, achieving motion correction within a reasonable time frame is not feasible with the current PRC method. Nevertheless, it is reasonable to expect improvements in diagnostic quality by integrating such combined corrections. This limitation stands as a significant constraint of this study.

This study was based on data from a single center. The reported SNR, CNR, and coefficient of variation may vary if different PET/CT or PET/MR systems are used. The limited size of our patient cohort constrains the capacity to fully evaluate the impact of STD + PRC on diagnostic quality. Nevertheless, it is of significant interest that STD + PRC demonstrated improved image quality. Another limitation is the need for a custom normal database to analyze STD + PRC, as special databases may not be clinical practice in some centers. Finally, the time required for reconstructions, approximately 6 h per MPI for this prototype implementation of the code (or 12 h per rest/stress scan per patient), may hinder the immediate clinical transition of the proposed PRC method. We expect that an optimized implementation of this prototype correction into the conventional reconstruction kernels may reduce the reconstruction time significantly, comparable to that for STD (3–4 min when using non-graphics processing unit–accelerated protocols).

## CONCLUSION

This study demonstrated the usefulness of retrospective PRC in routine clinical ^82^Rb PET MPI. The proposed PRC enhanced the diagnostic quality of the images by improving both SNR and CNR. Therefore, PRC can be recommended for routine clinical use, particularly as the reconstruction time may be shortened if the implementation of the corrections is optimized.

## DISCLOSURE

This project received funding from the European Union’s Horizon 2020 research and innovation program under grant agreements 670261 (ERC Advanced Grant) and 668532 (Click-It), the Lundbeck Foundation, the Novo Nordisk Foundation, the Innovation Fund Denmark, the Danish Cancer Society, the Arvid Nilsson Foundation, the Neye Foundation, the Research Foundation of Rigshospitalet, the Danish National Research Foundation (grant 126), the Research Council of the Capital Region of Denmark, the Danish Health Authority, the John and Birthe Meyer Foundation, and the Research Council for Independent Research. Andreas Kjaer is a Lundbeck Foundation Professor. Financial support was received from Siemens Medical Solutions USA, Inc. Vladimir Panin, Maurizio Conti, Sven Zuehlsdorff, Jorge Cabello, and Deepak Bharkhada are full-time employees at Siemens Medical Solutions, Inc. No other potential conflict of interest relevant to this article was reported.

## Notes

KEY POINTS **QUESTION:** Is it possible to retrospectively correct PRC on routine ^82^Rb PET images, and what are the clinical implications of these corrections? **PERTINENT FINDINGS:** PRC was successfully tested in 116 ^82^Rb PET acquisitions, and significant improvements for SNR and CNR were reported. The diagnostic quality of the images was verified against FFR, proving that PRC did not challenge the well-established diagnostic quality of ^82^Rb PET imaging. **IMPLICATIONS FOR PATIENT CARE:** The significantly improved image contrast from adding PRC is key to visually interpreting ^82^Rb PET images in clinical routine and will help to identify and verify perfusion defects.
